# 553. Outcomes in Patients Positive for Severe Acute Respiratory Syndrome Coronavirus 2 (SARS-CoV-2) Infection After Treatment with Monoclonal Antibody Therapy (MAT) in the Outpatient Setting

**DOI:** 10.1093/ofid/ofab466.751

**Published:** 2021-12-04

**Authors:** Courtney Nichols, Mark Lustberg, Mohammad Mahdee Sobhanie, Joy Lehman, Erica E Reed, Nicholas E Kman, Mark Conroy, Michael Dick, James N Allen, Jonathan Parsons, Carlos Malvestutto

**Affiliations:** 1 OSU Wexner Medical Center, Columbus, Ohio; 2 The Ohio State University, Columbus, Ohio; 3 The Ohio State University Wexner Medical Center, Columbus, OH; 4 Ohio State University Wexner Medical Center, Columbus, Ohio; 5 The Ohio State University College of Medicine, Columbus, Ohio; 6 Ohio State University, Columbus, Ohio

## Abstract

**Background:**

Monoclonal antibody therapy (MAT) was granted Emergency Use Authorization (EUA) by the U.S. Food and Drug Administration for treatment of mild to moderate coronavirus disease 2019 (COVID-19) in adults with positive SARS-CoV-2 viral testing and at high risk for progression to severe COVID-19 with up to 10 days of symptoms. This study assessed the impact of MAT on COVID-19-related ER visits, admissions, and mortality for patients diagnosed with COVID-19.

**Methods:**

This was a single-center, retrospective study at The Ohio State University Wexner Medical Center to compare COVID-19-related ER visits, admissions, and mortality at 30 days after receiving MAT in the outpatient setting with either bamlanivimab or casirivimab-imdevimab in adult patients diagnosed with SARS-CoV-2 between November 16, 2020 and February 2, 2021. Outcomes in patients who received MAT were compared to those of a control group of patients diagnosed with COVID-19 in the outpatient setting from May 16, 2020 through November 15, 2020 who would have qualified for MAT through EUA criteria had it been available. Statistical analysis used logistic regression analysis with backward selection to determine the odds ratios (OR) and the 95% confidence interval to evaluate the relationship between patient clinical characteristics and outcomes.

**Results:**

This study cohort included 1,944 patients, including 943 who received MAT and 1,001 in the control group. The MAT group included 658 who received bamlanivimab and 285 who received casirivimab-imdevimab. Patients who received MAT compared to the control group had a lower rate of COVID-19 related ER visits (3.3% vs 7.4%, p = < 0.0001) and hospital admissions (4.0% vs 7.8%, p = < 0.0001). No statistically significant difference was seen in mortality between the MAT group (0.5%) and control group (1.1%, p = 0.17). After accounting for potential confounders, the difference between the monoclonal antibody and control groups remained significant for ER visits and hospital admissions as reflected in the table.

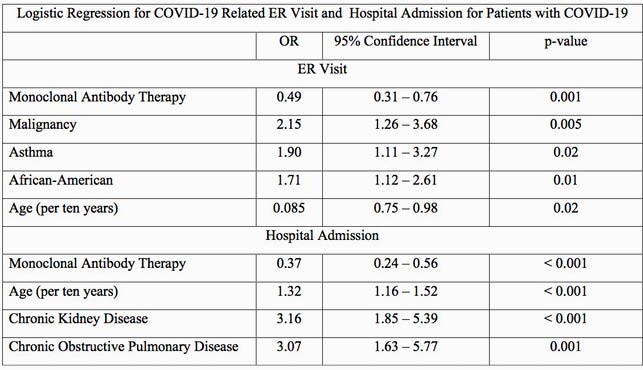

**Conclusion:**

Patients who received MAT for COVID-19 in the outpatient setting had a lower rate of COVID-19-related 30 day ER visits and hospitalizations compared to those who did not receive MAT, adjusting for potential confounders.

**Disclosures:**

**Mohammad Mahdee Sobhanie, M.D.**, **Regeneron** (Scientific Research Study Investigator)**Regeneron** (Scientific Research Study Investigator, Was a sub-investigator for Regeneron 2066 and 2069) **Carlos Malvestutto, M.D.**, **Lilly** (Scientific Research Study Investigator)**Regeneron Inc.** (Scientific Research Study Investigator)**ViiV Healthcare** (Advisor or Review Panel member)

